# A new dye uptake assay to test the activity of antibiotics against intracellular *Francisella tularensis*

**DOI:** 10.3389/fcimb.2014.00036

**Published:** 2014-03-18

**Authors:** Vivien Sutera, Yvan Caspar, Sandrine Boisset, Max Maurin

**Affiliations:** ^1^Laboratoire de Bactériologie, Département des Agents Infectieux, Institut de Biologie et de Pathologie, Centre Hospitalier Universitaire GrenobleGrenoble, France; ^2^Laboratoire Adaptation et Pathogénie des Micro-Organismes, Université Joseph Fourier-Grenoble 1Grenoble Cedex 9, France; ^3^CNRS, UMR 5163Grenoble, France

**Keywords:** tularaemia, *Francisella tularensis*, dye uptake assay, antibiotic activity, intracellular infection

## Abstract

*Francisella tularensis*, a facultative intracellular bacterium, is the aetiological agent of tularaemia. Antibiotic treatment of this zoonosis is based on the administration of a fluoroquinolone or a tetracycline for cases with mild to moderate severity, whereas an aminoglycoside (streptomycin or gentamicin) is advocated for severe cases. However, treatment failures and relapses remain frequent, especially in patients suffering from chronic lymph node suppuration. Therefore, new treatment alternatives are needed. We have developed a dye uptake assay for determination of minimal inhibitory extracellular concentrations (MIECs) of antibiotics against intracellular *F. tularensis*, and validated the method by comparing the results obtained using a CFU-enumerating method. We also compared MIECs with MICs of the same compounds determined using a CLSI broth microdilution method. We tested the activity of 11 antibiotics against two clinical strains of *F. tularensis* subsp. *holarctica* isolated in France. Both strains displayed low MICs (≤1 μg/mL) to fluoroquinolones (ciprofloxacin, levofloxacin and moxifloxacin), gentamicin, doxycycline and rifampicin. Higher MICs (≥8 μg/mL) were found for carbapenems (imipenem and meropenem), daptomycin and linezolid. Erythromycin MICs were 4.0 and 16.0 μg/mL, respectively, for the two clinical strains. MIECs were almost the same with the two methods used. They were concordant with MICs, except for erythromycin and linezolid (respectively, four and eight times more active against intracellular *F. tularensis*) and gentamicin (four to eight times less active against intracellular *F. tularensis*). This study validated the dye uptake assay as a new tool for determination of the activity of a large panel of antibiotics against intracellular *F. tularensis*. This test confirmed the intracellular activity of first-line antibiotics used for tularaemia treatment, but also revealed significant activity of linezolid against intracellular *F. tularensis*.

## Introduction

Tularaemia is a zoonotic disease caused by the Gram-negative bacterium *Francisella tularensis*. Two subspecies are responsible for the majority of human infections, including *F. tularensis* subsp. *tularensis* in North America and *F. tularensis* subsp*. holarctica* throughout the Northern hemisphere. The latter subspecies is split into two biovars. Biovar I, naturally susceptible to erythromycin, is found in North America and Western Europe. Biovar II, naturally resistant to erythromycin, is found in Eastern Europe and Asia (Keim et al., 2007). *F. tularensis* is highly infectious for humans and has been classified as a category A bioterrorism agent by the CDC (Bossi et al., 2004).

Only a few antibiotic classes are effective to treat tularaemia patients. The aminoglycosides (streptomycin and gentamicin) are considered the reference treatment for severe forms of the disease (Hepburn and Simon, 2008). For mild to moderate tularaemia cases, fluoroquinolones (ciprofloxacin, levofloxacin) and tetracyclines (doxycycline) are advocated as first-line drugs (Tärnvik and Chu, 2007). All these antibiotics have side effects and their use should be restricted especially in pregnant women (Dentan et al., 2013). Moreover, antibiotic treatments using a tetracycline or a fluoroquinolone are associated with high rates of failure and relapse (Johansson et al., 2001; Perez-Castrillon et al., 2001). Finally, high-level resistance to macrolides, tetracyclines and fluoroquinolones was easily selected *in vitro* in *F. tularensis* (La Scola et al., 2008; Gestin et al., 2010; Loveless et al., 2010; Sutera et al., 2014), which raises some concern about the misuse of resistant strains in the bioterrorism context or the possibility of *in vivo* selection of such resistance in tularaemia patients.

*F. tularensis* is a slow growing, facultative intracellular bacterium. It replicates in the cytoplasm of macrophages (Anthony et al., 1991; Chong and Celli, 2010) and non-phagocytic cells (Hall et al., 2007). Both cell types are involved in tularaemia patients, especially in the lower airways in patients suffering from pneumonia (Horzempa et al., 2010). A number of techniques have been developed to test the activity of antibiotics against intracellular pathogens. In most studies, the intracellular activity of the tested antibiotic is evaluated by measuring the viable bacterial counts (VBCs) after antibiotic exposure compared to an untreated control. VBCs are usually determined using the colony forming unit (CFU)-enumeration methodology (Segreti et al., 1996; Wright Valderas and Barrow, 2008). Because this technique is fastidious and time consuming and not adapted for microorganisms growing exclusively in eukaryotic cells, methods based on DNA quantification using quantitative real-time PCR technology (Boulos et al., 2004) or immunofluorescent-antibody testing (Ives et al., 1997) have been proposed. The intracellular growth of bacteria can also be deduced from their cytotoxic effect in eukaryotic cell culture systems (Edouard and Raoult, 2013), especially using a simple dye uptake assay, as previously described for strict intracellular bacteria such as *Rickettsia* species (Rolain et al., 1998). This technique is based on the capacity for live cells to internalize a vital dye such as neutral red (Borenfreund and Puerner, 1985). In this system, the activity of an antibiotic is deduced from its potential to prevent cytotoxic effects by inhibiting bacterial multiplication.

In this study, we adapted the dye uptake assay to evaluate the activity of several antibiotics against two clinical strains of *F. tularensis* subsp. *holarctica*. Our first goal was to demonstrate that this simple technique gives equivalent results compared to the VBCs method. Because the dye uptake assay is much easier to perform, it allowed us to screen the intracellular activity of a large number of antibiotic compounds against several strains of *F. tularensis*. This work could facilitate the search for new treatment alternatives for tularaemia, as well as the detection of acquired resistances to available antibiotics.

## Materials and methods

### Bacterial strains and cell line

Two clinical strains of *F. tularensis* subsp. *holarctica* were used: Ft6 isolated in 2007 from a blood culture and Ft24 isolated in 2009 from an axillary lymphadenopathy. Both strains were identified to the subspecies level by sequencing the intergenic region located between 16S and 23S RNA encoding genes (Maurin et al., 2011). They were kept frozen in cryotubes (MastDiagnostic, Amiens, France) at −80°C. They were grown in a biosafety level 3 laboratory, using chocolate agar supplemented with Polyvitex® (CHA-PVX medium, bioMérieux, Marcy l'Etoile, France) incubated at 37°C in a 5% CO_2_-enriched atmosphere.

We used three control strains for MIC determination: *Escherichia coli* ATCC25922, *Pseudomonas aeruginosa* ATCC27853 and *Staphylococcus aureus* ATCC29213. They were grown on Columbia medium supplemented with 5% sheep blood (COS medium, bioMérieux) incubated 24 h at 37°C with 5% CO_2_.

For the cell system, we used the human pulmonary diploid fibroblastic cells MRC-5 (RD Biotech, Besançon, France). Cell monolayers were grown in Minimum Essential Medium (MEM, Gibco®, Life Technologies, Saint Aubin, France) supplemented with 10% decomplemented foetal calf serum (FCS, Gibco), at 37°C, in a 5%CO_2_-enriched atmosphere. These fibroblastic cells are strictly adhesive and stop their multiplication when at confluence, which enables cells proliferation control. Moreover, this model has already been used for susceptibility testing of other pathogens such as *Legionella pneumophila* (Segreti et al., 1996) or *Tropheryma whipplei* (Boulos et al., 2004). Finally, *F. tularensis* subsp. *holarctica* strains were able to efficiently infect and proliferate in this model (*cf*. Result section).

### Antibiotics

We used ciprofloxacin (Panpharma, Fougères, France), levofloxacin (Fresenius kabi, Sèvres, France), moxifloxacin (Bayer, Puteaux, France), imipenem (Panpharma), meropenem (Panpharma), daptomycin (Novartis, Rueil-Malmaison, France), doxycycline (Sigma-Aldrich, Lyon, France), rifampicin (Sanofi-Aventis, Paris, France), gentamicin (Panpharma), linezolid (Pfizer, Paris, France) and erythromycin (Fluka, Lausanne, Switzerland). Stock solutions of these 11 antibiotics were prepared in sterile distilled water for gentamicin, ciprofloxacin and erythromycin, and in 0.45% sodium chloride solution for the other antibiotics, and kept frozen at −80°C until used.

### MIC determination

MICs were determined using a microdilution method in Mueller-Hinton (MH) broth (bioMérieux) supplemented with 2% PolyViteX®, using a CLSI methodology (Clinical and Laboratory Standards Institute, 2009).

Briefly, each antibiotic was diluted in MH-2%PVX to obtain twofold serial concentrations (Table 1), and 75 μL of each suspension was dispensed in one well of 96-well microtiter plates. An equal volume of a 10^6^ bacterial suspension was added to each well. After incubation of the plates for 48 h at 37°C, the lowest antibiotic concentration inhibiting visible bacterial growth was recorded as the MIC. All experiments were run in duplicate. The reference strains *E. coli* ATCC25922, *P. aeruginosa* ATCC27853 and *S. aureus* ATCC29213 were used as positive controls. Wells receiving only MH broth were used as negative controls.

**Table 1:** **Ranges of antibiotic concentrations tested for determination of MICs (broth microdilution method) and MIECs (dye uptake assay) for 11 antibiotics against *Francisella tularensis* subsp. *holarctica***.

**Antibiotics**	**Antibiotic targets**	**MIC (μg/mL)**	**MIEC (μg/mL)**
Gentamicin	Ribosome	0.032–16	0.064–32
Ciprofloxacin	Type II topoisomerases	0.001–0.5	0.004–2.0
Levofloxacin	Type II topoisomerases	0.001–0.5	0.004–2.0
Moxifloxacin	Type II topoisomerases	0.001–0.5	0.004–2.0
Doxycycline	Ribosome	0.032–16	0.032–16
Erythromycin	Ribosome	0.25–128	0.25–128
Imipenem	Cell wall	0.25–128	0.25–128
Meropenem	Cell wall	0.25–128	0.25–128
Linezolid	Ribosome	0.064–32	0.064–32
Rifampicin	RNA polymerase	0.004–2.0	0.016–8.0
Daptomycin	Cytoplasmic membrane	0.5–256	0.5–256

*MIC, minimum inhibitory concentration; MIEC, minimum inhibitory extracellular concentration*.

### MIEC determination

The MRC-5 cells were prepared in MEM-10% FCS at a concentration of 6 × 10^5^ cells/mL. This cell suspension was dispensed (100 μL per well) in flat-bottom 96-well microtiter plates, and incubated 16 h at 37°C in a 5% CO_2_-enriched atmosphere to obtain confluent cell monolayers. In parallel, a 1-McFd standard suspension of each *F. tularensis* strain tested was grown for 24 h in brain-heart infusion broth (BHI, bioMérieux) supplemented with 2% PVX, at 37°C, in 5%CO_2_. For each strain, the bacterial suspension obtained was adjusted to 0.5 McF standard by adding BHI-2%PVX, and further diluted in MEM-10% FCS to achieve a bacterial inoculum of 1.2 × 10^5^ bacteria/50 μL of medium (i.e., 2.4 × 10^6^ bacteria/mL). The cell monolayers were infected by replacing the supernatant with 50 μL of the previously prepared bacterial suspension. The plates were incubated at 37°C, in 5% CO_2_ for 3 h to allow internalization of bacteria into MRC-5 cells. Cell monolayers were then washed in pH 7.2 sterile phosphate buffer saline (PBS, Gibco) and re-incubated for 1 h (37°C, 5% CO_2,_)in MEM-10% FCS medium containing 5 μg/mL of gentamicin in order to eliminate extracellular bacteria. At that time, the intracellular bacterium inoculum (referred to as the primary intracellular inoculum) was determined using the CFU-enumeration method (see below). After 3 additional washes with PBS, infected MRC-5 monolayers were incubated (37°C, 5% CO_2_) in MEM-10% FCS medium containing twofold serial concentrations of the tested antibiotic (Table 1). MIECs were read after 1 or 5 days incubation of the plates at 37°C, in 5% CO_2_for the CFU method and the dye uptake assay, respectively. All assays included a *F. tularensis* positive growth control (infected MRC-5 cells with no antibiotic) and two negative controls (uninfected MRC-5 with no antibiotic or with the antibiotic at the maximum concentration tested). The activity of antibiotics against intracellular *F. tularensis* was then evaluated in parallel using two methods: the dye uptake assay and CFU counting. All experiments were run in duplicates to confirm results.

#### Dye uptake assay

**Figure 1:**
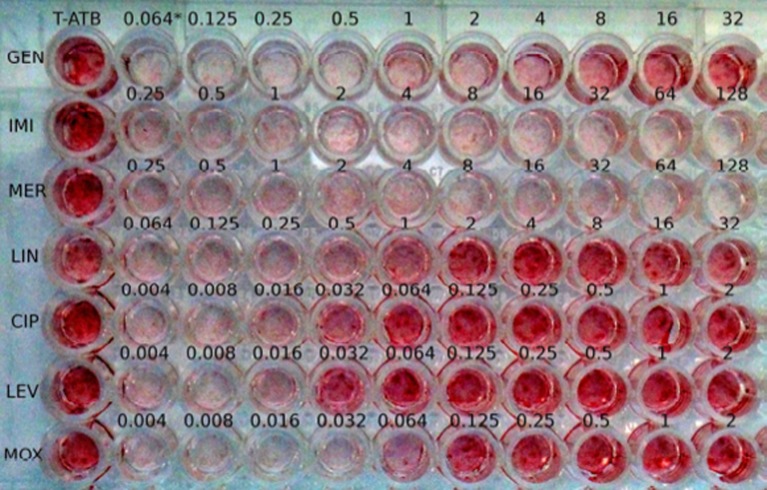
**Image of a dye uptake assay for the Ft24 strain**. ^*^, antibiotic concentrations in the well (μg/mL); T-ATB, uninfected MRC-5 monolayer incubated with the highest concentration of the antibiotic tested; GEN, gentamicin; IMI, imipenem; MER, meropenem; LIN, linezolid; CIP, ciprofloxacin; LEV, levofloxacin; MOX, moxifloxacin.

After incubation of the plates, the cell supernatants were removed and replaced with 50 μL of 0.15% neutral red dye (Sigma-Aldrich) in PBS, pH 5.5. The plates were incubated 1 h at 37°C in 5% CO_2_ to allow penetration of the dye into the cells. The excess dye was then removed by three washes in PBS, pH 6.5. The red staining of cell monolayers was visually evaluated in comparison to positive (T+, MRC-5 monolayer infected at MOI 200:1 without antibiotics) and negative controls (T-ATB, uninfected MRC-5 monolayer incubated with the highest concentration of the antibiotic tested). A staining score of 1 corresponded to complete lysis of the cell monolayer, i.e., T+ control. A staining score of 4 corresponded to full preservation of the cell monolayer, i.e., T-ATB control. Scores 2 and 3 corresponded to intermediate color intensities (Figure 1). The MIEC was defined as the minimum extracellular concentration of the antibiotic tested allowing prevention of a *F. tularensis* cytotoxic effect (staining score of 4).

#### CFU counts

After incubation of the plates, the cell supernatants were removed and replaced with 200 μL of 1% saponin solution (ProLabo®, Leuven, Belgium). The plates were incubated 15 min at room temperature to allow disruption of the eukaryotic cell membranes and release of intracellular bacteria into the cell supernatant. After homogenisation, 1, 10, and 100 μL of the bacterial suspension of each well were plated on CHA-PVX media. CFU counts were determined after 48 h incubation of the CHA-PVX plates at 37°C, in 5% CO_2_. The same procedure was used to determine the primary intracellular bacterial inoculum, as mentioned above. Thus the activity of antibiotics was deduced from their capacity to completely inhibit bacterial growth, i.e., CFU counts after antibiotic exposure ≥ CFU counts of the primary intracellular bacterial inoculum.

## Results

### Dye uptake assay validation

**Figure 2:**
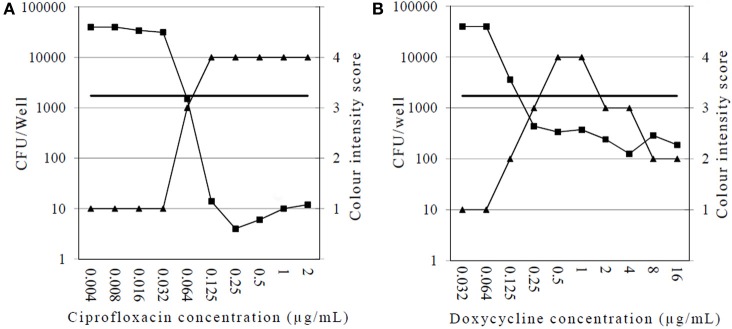
**Comparison of results obtained with the CFU enumeration and dye uptake assays for intracellular activity of ciprofloxacin (A) and doxycycline (B) against *F. tularensis* subsp. *holarctica* stain Ft6**. Squares, CFU counts 24 h post-infection (CFU/well); triangles, dye uptake assay (score 1–4); horizontal line, 1 h post-infection intracellular bacterial load (CFU/well) as determined by the CFU-enumeration method (results of one experiment).

We first tested the activity of ciprofloxacin and doxycycline against intracellular *F. tularensis* Ft6 strain, using both the CFU and dye uptake assays (Figure 2). For drug-free controls, the mean increase in intracellular bacterial loads after 24 h incubation of cultures was between 2.41 and 3.43 log CFU/well (data not shown). As for ciprofloxacin (Figure 2A), the CFU method determined the MIEC to be 0.125 μg/mL. The same method also revealed that ciprofloxacin induced a two-log reduction of bacterial loads at concentrations above the MIEC. The dye uptake assay showed complete destruction of the cell monolayers after 5 days incubation of cultures (score = 1) at ciprofloxacin concentrations up to 0.032 μg/mL, a score of 3 at 0.064 μg/mL, and a score of 4 for concentrations ≥ 0.125 μg/mL. Thus, ciprofloxacin MIEC was the same for the dye uptake and CFU count assays. For doxycycline (Figure 2B), the MIECs were 0.25 μg/mL and 0.5 μg/mL using the CFU and dye uptake assays, respectively. Doxycycline only induced a lower (<1 log) reduction in bacterial counts after 24 h incubation. Also, a reduction in the dye uptake scores was observed for doxycycline concentrations ≥2 μg/mL, suggesting a toxic effect of this compound against MRC-5 cells at these concentrations.

### MICs and MIECs of 11 antibiotics against *F. tularensis* Ft6 and Ft24 strains

Both strains displayed low MICs for gentamicin, fluoroquinolones (ciprofloxacin, levofloxacin and moxifloxacin), doxycycline and rifampicin (Table 2). Erythromycin was less effective, with four times higher MIC for the Ft24 strain compared to the Ft6 strain. The carbapenems (imipenem, meropenem) and daptomycin had no inhibitory activity against *F. tularensis*. Similar MIC and MIEC values were obtained for fluoroquinolones, doxycycline and rifampicin. Gentamicin was less effective against intracellular *F. tularensis*, with MIECs four to eight times higher than MICs. In contrast, erythromycin and linezolid displayed improved activity against intracellular *F. tularensis*. MIECs were four to eight times lower than MICs for erythromycin and eight times lower than MICs for linezolid.

**Table 2:** **Activities of 11 antibiotics against two clinical strains (Ft6 and Ft24) of *F. tularensis* subsp. *holarctica* determined using a broth microdilution method and a dye uptake assay, respectively**.

	**MIC (μg/mL)**		**MIEC (μg/mL)**	
	**Ft6**	**Ft24**	**Ft6**	**Ft24**
Gentamicin	0.25	0.5	2	2
Ciprofloxacin	0.032	0.032	0.064	0.064
Levofloxacin	0.064	0.064	0.064	0.064
Moxifloxacin	0.125	0.125	0.125	0.125
Doxycycline	0.5	1	0.5	0.5
Erythromycin	4	16	1	2
Imipenem	>128	>128	>128	>128
Meropenem	>128	>128	>128	>128
Linezolid	8	8	1	1
Rifampicin	0.5	0.5	0.5	0.5
Daptomycin	>256	>256	>256	>256

*MIC, minimum inhibitory concentration; MIEC, minimum inhibitory extracellular concentration*.

## Discussion

We adapted a dye uptake assay previously used to test the antibiotic susceptibilities of strict intracellular pathogens (Rolain et al., 1998) to investigate the activity of antibiotics against the intracellular form of the facultative intracellular bacterium *F. tularensis*. This test is safer than traditional CFU-based assays because it does not need manipulation of large quantities of culture dishes of this highly infectious agent. Although the test should be performed in a biosafety level 3 laboratory, the intracellular activity of several compounds against a large panel of type A and type B *F. tularensis* strains could be easily achieved. We first validated this new assay by comparing the results obtained for two antibiotics against two clinical strains of *F. tularensis* subsp. *holarctica* when using either the dye uptake assay or a traditional method of VBC determination using CFU methodology. We found a high correlation between MIECs determined using both methods. This was not unexpected since, in our model, *F. tularensis* multiplication led to complete lysis of cell monolayers. In contrast, inhibition of intracellular bacterial growth prevented *F. tularensis* cytolytic effects. Complete monolayer lysis was revealed using the live stain neutral red. This molecule is commonly used for cytotoxicity (Borenfreund and Puerner, 1985) or vacuolation assays (Cover et al., 1991), as it rapidly accumulates in lysosomes after penetration in viable cells by diffusion through the cytoplasmic membrane. It has been previously shown that the presence of weak bases (Ohkuma and Poole, 1981) or bacterial compound (Cover et al., 1991) influences vacuoles production level. Crystal violet has been used for similar experiments with the advantage that this stain binds to chromatin and thus color intensity may not vary according to the antibiotic used (Brasaemle and Attie, 1988). However, we observed an increase in neutral red uptake in cells exposed to antibiotics facilitating interpretation of results due to higher contrast between lysed and unlysed monolayers (Figure 1). For standardization, we framed each assay with controls used as visual cut-off for independent interpretation for each antibiotic (i.e., Materials and Methods section).

We then evaluated the extracellular and intracellular activity of 11 antibiotic compounds against the same two clinical strains of *F. tularensis*, respectively, using a CLSI broth microdilution method and the dye uptake assay developed. The broth microdilution method for antibiotic susceptibility testing of *F. tularensis* remains fastidious and the results are poorly predictive of the clinical situation for some antibiotics (Valade et al., 2008). Numerous controls are also needed, such as the use of non-fastidious control strains to check antibiotic activity in more standardized conditions. MIC results were consistent with previous studies. As expected, the carbapenems, which belong to the beta-lactams family, were not effective against *F. tularensis* (Georgi et al., 2012). Both strains were susceptible to antibiotics used as first-line treatment of tularaemia, including three fluoroquinolone compounds, doxycycline and gentamicin (Urich and Petersen, 2008; Valade et al., 2008). Rifampicin was also highly effective against *F. tularensis in vitro*. Erythromycin was less effective, although we tested type B biovar I strains of *F. tularensis*, which was consistent with previous studies (Gestin et al., 2010; Georgi et al., 2012). The new compounds daptomycin (a lipopeptide) and linezolid (an oxazolidinone) were either ineffective or poorly effective against extracellular *F. tularensis*. To our knowledge, linezolid has only been tested against *Francisella* sp. strains using MIC test strips, with inconsistent results between studies. MIC ranges were 2–16 μg/mL for North American strains tested using Mueller-Hinton supplemented with 1% IsovitaleX (Johansson et al., 2002) and 0.5–2.0 μg/mL for Turkish strains from central Anatolia tested using glucose/cysteine blood agar (GCBA) plates supplemented with 9% sheep blood (Yeşilyurt et al., 2011), whereas the MIC_90_ was 32 μg/mL for Hungarian strains tested using modified Francis agar plates (Kreizinger et al., 2012). These discrepancies may represent true differences in linezolid susceptibilities among *F. tularensis* strains of different geographic origin, but may also reflect poor standardization of the methods used. Valade et al. (2008) previously demonstrated that MIC test strips gave different results when using different agar media, and that the results obtained with this method were poorly correlated to those obtained using the reference agar dilution method, especially for nalidixic acid and rifampicin.

We found ciprofloxacin and doxycycline had similar inhibitory activity against extracellular and intracellular *F. tularensis*. These two antibiotic classes are concentrated within eukaryotic cells (Hof, 2003) and are used as reference treatments for infectious diseases caused by intracellular pathogens (Rolain et al., 1998; Wright Valderas and Barrow, 2008). However, using the CFU method, we found a more pronounced bactericidal effect of ciprofloxacin as compared to doxycycline against *F. tularensis* strains grown in MRC-5 cells, which confirms previously published data using macrophage-like cells (Maurin et al., 2000). These findings are consistent with the current recommendation of the use of ciprofloxacin and doxycycline as first-line treatment of tularaemia (Johansson et al., 2000; Scheftel et al., 2010; Maurin et al., 2011). In our model, the aminoglycoside gentamicin was four to eight times less effective against the intracellular form of *F. tularensis*. Previous experiments have shown that gentamicin displays a bactericidal activity against intracellular *F. tularensis*, but prolonged exposure of infected cells to this antibiotic is needed because of its slow penetration and concentration within eukaryotic cells (Maurin and Raoult, 2001). Whereas streptomycin was considered the referenced treatment of tularaemia (Tärnvik and Chu, 2007), gentamicin has been recently associated with treatment failures and relapses (Kaya et al., 2012).

Regarding antibiotics not currently recommended for treatment of tularaemia, three situations were observed. Rifampicin displayed similar extracellular and intracellular activities. This antibiotic is not used for treatment of tularaemia because of concern about selection of resistant mutants (Bhatnagar et al., 1994). Daptomycin and the carbapenems were not effective against the extra- and intracellular forms of the two *F. tularensis* strains tested. The beta-lactams are usually considered unreliable for treatment of tularaemia (Cross and Jacobs, 1993). Although *F. tularensis* may harbor a class A beta-lactamase (Antunes et al., 2012), mechanisms of resistance to carbapenems in this species need further investigation. To our knowledge, susceptibility to imipenem has been reported only for three biovar II strains of *F. tularensis* subsp. *holarctica* (including the LVS strain) (Tomaso et al., 2005). Lee et al. (1991) reported a case of tularaemia with favorable progression after 14-day treatment with imipenem. Finally, erythromycin and linezolid were more active when tested in the MRC-5 cell system. MIECs were eight times lower than MICs, suggesting that these compounds could concentrate in the intracellular compartment of *F. tularensis* multiplication. The same observation was previously reported for azithromycin and the LVS strain of *F. tularensis* (Ahmad et al., 2010). Erythromycin, like other macrolides, can concentrate within acidic compartments of eukaryotic cells (especially lysosomes) because of their low base nature (Carlier et al., 1987). However, their intracellular activity may be reduced owing to their protonation at acidic pH (Goldman et al., 1990). The macrolides are not considered a safe alternative for tularaemia patients (Enderlin et al., 1994), but recent case reports indicate that azithromycin may be useful in pregnant women with mild disease caused by type B biovar I strains of *F. tularensis* (Dentan et al., 2013). More surprisingly, linezolid displayed significant activity against intracellular *F. tularensis in vitro*. This oxazolidinone is currently used for treatment of infections caused by multi-drug-resistant Gram-positive bacterial species, such as *S. aureus, Streptococcus pneumoniae* and *Enterococcus* sp. Interestingly, an additive effect of the combination of linezolid and gentamicin was reported against *S. aureus* (Grohs et al., 2003). Linezolid is not active against aerobic Gram-negative bacteria such as enterobacterial and *Pseudomonas* sp. (Leclercq, 2010). On the other hand, linezolid did not accumulate in THP-1 human macrophage cells (Lemaire et al., 2011). Thus, the mechanism of action of linezolid against intracellular *F. tularensis* should be further investigated.

In conclusion, we adapted a dye uptake assay in order to evaluate the activity of antibiotics against intracellular *F. tularensis*. This test would facilitate screening of the activity of new compounds against this fastidious, facultative intracellular bacterium in the search for new therapeutic alternatives for tularaemia. Also, because the proposed technique is much easier to perform than the traditional CFU methodology, it may help standardize antibiotic susceptibility testing for *F. tularensis* strains. Finally, this study highlights the potential usefulness of linezolid as a therapeutic alternative for tularaemia patients, especially in case of failure or relapses after administration of current first-line antibiotics. *In vitro* results obtained with this drug warrant further investigation in animal models.

## Author contribution

Research project design: Vivien Sutera and Max Maurin. Experiments: Vivien Sutera, Yvan Caspar and Sandrine Boisset. Writing: Vivien Sutera and Max Maurin.

### Conflict of interest statement

The authors declare that the research was conducted in the absence of any commercial or financial relationships that could be construed as a potential conflict of interest.
